# Challenges and Treatment Strategies in Elderly Patients with Inflammatory Bowel Disease: A Systematic Review and Narrative Synthesis

**DOI:** 10.3390/jpm16020059

**Published:** 2026-01-23

**Authors:** John K. Triantafillidis, Konstantinos Malgarinos, Georgia Kontrarou, Emmanouil Kritsotakis, Victoria Polydorou, Konstantinos Pantos, Konstantinos Sfakianoudis, Agni Pantou, Anastasios Karandreas, Manousos M. Konstandoulakis, Apostolos E. Papalois

**Affiliations:** 1 IBD Unit, “METROPOLITAN GENERAL” Hospital, 264 Mesogeion Avenue, 15562 Athens, Greece; jktrian@gmail.com (J.K.T.); konstantinosmalgarinos@gmail.com (K.M.); gkontrarou@yahoo.gr (G.K.); 2 Hellenic Society of Gastrointestinal Oncology, 354, Iera Odos Str., 12461 Athens, Greece; mkonstad@med.uoa.gr; 3 2nd Department of Surgery, Aretaieion University Hospital, School of Medicine, University of Athens, 76 Vas. Sophias Av., 11528 Athens, Greece; ekritsotakis2020@gmail.com; 4 School of Medicine, European University Cyprus, 6 Diogenous St. Egkomi, 1516 Nicosia, Cyprus; v.polydorou@euc.ac.cy (V.P.); surgeon1@otenet.gr (A.K.); 5 Centre for Human Reproduction, Genesis Athens Clinic, 14-16, 15232 Athens, Greece; info@pantos.gr (K.P.); sfakianosc@yahoo.gr (K.S.); agni.pantos@gmail.com (A.P.)

**Keywords:** inflammatory bowel disease, ulcerative colitis, Crohn’s disease, elderly, treatment, biologic agents

## Abstract

**Introduction**: The proportion of elderly patients with IBD is steadily increasing due to the aging population and improved survival. Patients in this age group present specificities in diagnosis and treatment, particularly regarding the use of biological agents, where immunosenescence, multimorbidity, and polypharmacy affect the precise assessment of benefit and risk. **Aim**: This systematic review, which was conducted in accordance with the PRISMA 2020 statement, aims to synthesize available data on the epidemiology, clinical characteristics, and therapeutic management of IBD in the elderly, with emphasis on the most recent data and practical guidelines for the use of biological therapies. **Methods**: A systematic search of PubMed, Scopus, and Embase was conducted. A total of 40 studies were included, comprising 5 randomized controlled trials, 15 prospective cohort studies, and 20 retrospective observational studies. Eligible studies included randomized controlled trials, observational cohort studies, and population-based analyses. Given substantial clinical and methodological heterogeneity, findings were synthesized narratively. Data on demographics, disease phenotype, comorbidities, and treatment outcomes were extracted and analyzed. In addition, a narrative synthesis of major randomized trials of biologic therapies, recent guidelines, and data from prospective studies and patient registries was performed with a focus on safety and real-world outcomes in the elderly. Risk of bias was assessed using the Newcastle–Ottawa Scale (NOS) and the Cochrane Risk of Bias tool. **Results**: The majority of included studies (85%) were found to have a low to moderate risk of bias, providing a reliable basis for the synthesis. Data show an increasing incidence of IBD in the elderly, often with a milder clinical course and a higher ratio of UC to CD. Multimorbidity and polypharmacy are significant challenges that increase the risk of adverse events. Although classic therapies remain effective, in many cases, a lower threshold is required to initiate advanced therapies, such as biologic agents. Anti-tumor necrosis factor (anti-TNF) agents, as well as biologics with alternative mechanisms of action such as vedolizumab (α4β7 integrin antagonist) and ustekinumab (interleukin-12/23 inhibitor), represent key therapeutic options in elderly patients with IBD. These biologic factors have efficacy comparable to that in younger patients and are considered attractive options due to reduced systemic immunosuppression and favorable safety profiles. JAK inhibitors are a practical option but are associated with an increased thromboembolic risk and require careful patient selection. Older age is associated with higher absolute rates of serious infections, hospitalizations, and, in some series, mortality. Individualized decision-making, including frailty assessment, vaccination coverage, infection control, and dose adjustments based on renal and hepatic function, is essential for optimal care. **Conclusions**: IBD in the elderly is a distinct clinical entity with unique challenges in diagnosis and management. A multidisciplinary approach and individualized treatment strategies are essential to ensure the balance between disease control and minimizing the risks associated with comorbidity and polypharmacy. Further research, including specifically designed clinical trials, is needed to optimize treatment and outcomes in this unique patient group.

## 1. Introduction

Inflammatory bowel diseases (IBD), which include Crohn’s disease (CD) and ulcerative colitis (UC), are chronic, relapsing inflammatory diseases of the gastrointestinal tract. Although the peak incidence is usually observed in young adulthood, in recent years, a second, less common “wave” of incidence has been observed in people over 60 years of age, known as elderly-onset IBD. This trend, combined with the continued increase in life expectancy, has resulted in a rapid rise in the proportion of elderly patients with IBD, whether these are newly diagnosed patients (late-onset IBD) or individuals with a long-term course of the disease entering geriatric age. Late-onset IBD is defined as diagnosis at the age of 60 years or older and should be clearly distinguished from long-standing IBD diagnosed earlier in life but persisting into older age. These two entities differ with regard to disease phenotype, comorbidity burden, risk of adverse events, and therapeutic decision-making. Late-onset IBD differs significantly from that in younger adults, both in terms of phenotypic characteristics (e.g., a smaller intestinal extent of disease in some series) and in the burden of comorbidities—cardiometabolic diseases, malignancies, frailty, and reduced physiological reserve. These factors affect diagnostic clarity (as symptoms may be confused with other gastrointestinal diseases) and make therapeutic decision-making much more complex [1,2,3].

A particular challenge is the selection and use of biologic agents, where efficacy must be weighed against the risks of infections, malignancies, and potential interactions with drugs used to treat comorbidities. In addition, polypharmacy and immunosenescence alter the safety profile of available therapies and affect the immune response to drugs and vaccinations.

The management of these patients is multifactorial and requires individualization, as the physician must balance the goals of disease remission and complication prevention while maintaining a low risk of adverse events. Treatment decisions should be made within a holistic, multidisciplinary framework, taking into accounts the patient’s vulnerability, functional status, and preferences [4,5].

This systematic review aims to provide a comprehensive and up-to-date picture of Late-onset IBD, highlighting epidemiological and clinical differences compared with younger patients, and critically evaluating the efficacy and safety of available therapeutic strategies. The aim is to highlight best practices and identify research gaps to guide future studies that improve the care of this vulnerable patient group.

## 2. Methods

This study was conducted in accordance with the PRISMA 2020 (Preferred Reporting Items for Systematic Reviews and Meta-Analyses) guidelines (Table S2). Also an unregistered study. A systematic literature search was conducted using PubMed (via pubmed.ncbi.nlm.nih.gov accessed on 1 September 2025), Scopus (via elsevier.com accessed 1 September 2025), and Embase (via elsevier.com accessed 1 September 2025) to identify relevant studies published between January 2000 and January 2025. No additional hand-searching or gray literature searches were undertaken. The search was limited to the aforementioned electronic databases. Supplementary searching of reference lists of included studies and forward citation searching was not performed as part of this review protocol. Studies were eligible if they included patients aged 60 years or older or provided extractable data for this age group.

Filters were applied for human studies published in English and publications from 2000 to 2025. The English language filter was applied as it is the primary language for international clinical evidence dissemination. The timeframe (2000–2025) was selected to capture the ‘biologic era’ of IBD management, ensuring that the synthesized data reflects current therapeutic standards and medications available in modern clinical practice

The inclusion criteria included evaluating only original research articles, systematic reviews, and meta-analyses that reported data on patients aged ≥60 years with a confirmed diagnosis of IBD. Exclusion criteria included case reports, conference abstracts, and publications in languages other than English. Two independent reviewers screened titles and abstracts to assess eligibility. The full texts of studies that met the inclusion criteria were then reviewed in detail for final selection. For each included study, information was extracted on study design, patient demographics, disease phenotype, treatment outcomes, and adverse events. Study quality was assessed using the Newcastle–Ottawa Scale (NOS) for observational studies. Any disagreements were resolved by consensus among the investigators.

Regarding data synthesis and analysis, the present review was organized as a structured narrative synthesis, which included three primary levels of analysis: Randomized controlled trials (RCTs) that determined the efficacy of biological therapies, such as GEMINI (vedolizumab), UNITI/IM-UNITI (ustekinumab), ACCENT/ACT (anti-TNF programs), and SONIC (combination therapy). Guidelines from international scientific societies (ECCO, AGA, BSG) regarding therapeutic practice and pre-treatment screening, and observational studies and patient registries (2015–2024) that reported age-stratified data on the efficacy and safety of biological therapies in the elderly. The decision to include this period was based on the significant evolution of the IBD therapeutic landscape during this decade, including the introduction of second-generation biologics (e.g., vedolizumab) and updated safety protocols specifically tailored for the geriatric population, which rendered older guidelines obsolete. Data were entered into a standardized extraction table, which included: study name and year, country of study, population size, mean age, therapeutic agent, dosage, duration of follow-up, primary outcomes, and specific findings for the elderly group. Data extraction was performed independently by two reviewers, with discrepancies resolved by consensus.

## 3. Results

**Figure 1:**
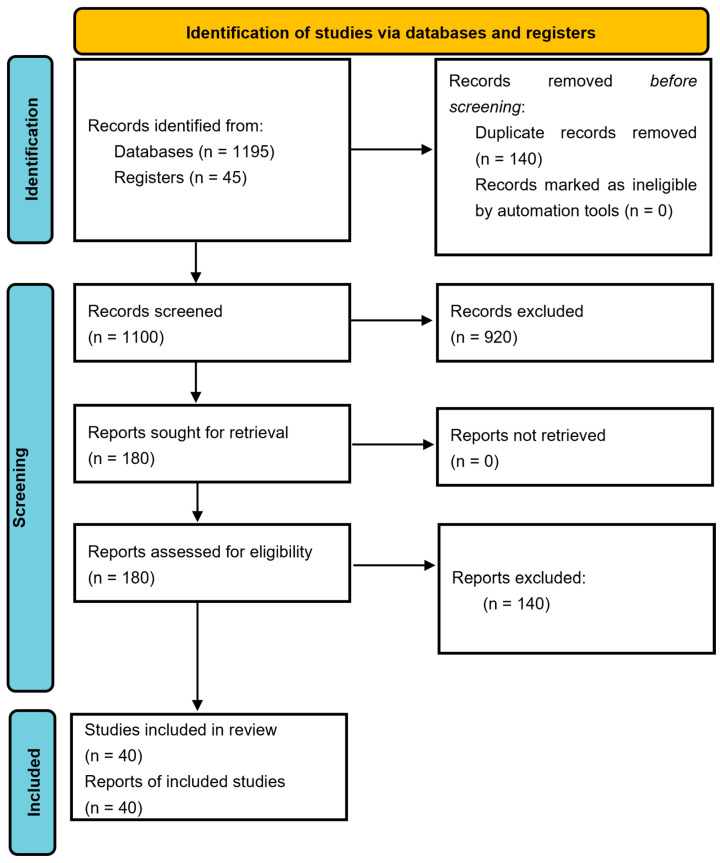
PRISMA 2020 flow-chart used in this study.

Figure 1 illustrates the PRISMA flow diagram summarizing study identification, screening, eligibility assessment, and final inclusion, and therefore reflects the results of the selection process rather than the methodological framework.

Quality assessment using the Newcastle–Ottawa Scale (NOS) for observational studies yielded an average score of 7.2/9, indicating high methodological quality. The RCTs assessed via the Cochrane tool showed a low risk of bias regarding outcome reporting, although 2 studies had an unclear risk regarding blinding of participants. Overall, the risk of bias was moderate, reflecting the predominance of retrospective and observational study designs, heterogeneous outcome definitions, and limited adjustment for confounding in several studies.

The results regarding the epidemiology (incidence and prevalence), disease phenotype, clinical characteristics, and treatment strategies (with emphasis on biological agents for IBD) in the elderly are described subsequently.

### 3.1. Epidemiology

#### Incidence and Prevalence

Late-onset IBD represents a substantial minority of all incident IBD cases, with prevalence steadily increasing as global populations age [6]. Between 1990 and 2019, the number of prevalent and incident cases, disability-adjusted life years (DALYs), and deaths attributed to Late-onset IBD rose markedly. Although age-standardized rates for incidence, prevalence, DALYs, and mortality showed a downward trend, the absolute disease burden increased significantly [7]. High-Socio-demographic Index (SDI) regions—including the Americas and Europe—maintained the greatest overall burden. In contrast, middle-SDI regions demonstrated the fastest rise in both prevalence and incidence, alongside the most rapid decline in DALYs and mortality.

Age-standardized prevalence, incidence, and DALY rates were highest in the 60–64-year age group, while mortality peaked among those aged 80–89 years. No significant sex differences were identified. These findings support the growing recognition of IBD in the elderly as a significant global public health concern, with considerable heterogeneity across regions and age strata [7].

In another study it was found that the global age-standardized incidence rate of IBD among individuals aged ≥60 years rose from 8.01 to 8.77 per 100,000 between 1990 and 2021 [8]. Lin et al. [9] estimated approximately 375,140 new IBD cases worldwide in 2021, corresponding to an age-standardized incidence rate of 4.45 per 100,000 for all ages. Danpanichkul et al. [10] further demonstrated that elderly-onset IBD accounted for roughly 11% of global incident cases in 2021. Across 167 countries, incidence rates increased most notably among females (annual percent change [APC] +0.06%) and the elderly (APC +0.14%), while remaining stable in males and the general population. In the United States, the same group of authors later observed that older-onset IBD constituted 15% of all IBD cases in 2021—a 3% rise since 2000. Between 2000 and 2021, the incidence (annual percent change 0.58%) and DALY rates (0.34%) increased, while prevalence remained stable. Notably, incidence rose more rapidly in older females compared with males [11]. Collectively, these data predict that by 2030, one-third of all IBD patients worldwide will be aged ≥60 years [12].

**Figure 2:**
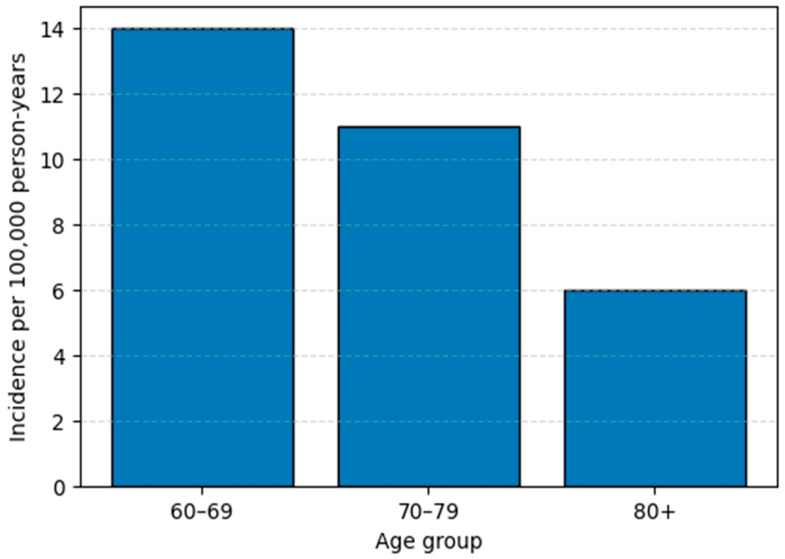
Age-specific incidence of Late-onset IBD. Incidence rates are presented per 100,000 person-years across different elderly age groups, illustrating the declining but clinically significant burden of IBD with advancing age. The figure highlights the epidemiological rationale for individualized management strategies in older patients.

Collectively, the age-specific incidence rates among Late-onset patients demonstrate that IBD remains a clinically relevant condition even in advanced age, with higher incidence observed in patients aged 60–69 years and a gradual decline in older age groups. This epidemiological pattern highlights the sustained disease burden in elderly populations and underscores the need for age-adapted management strategies (Figure 2).

In summary, Late-onset IBD accounts for approximately 10–15% of all new IBD diagnoses, with prevalence steadily rising in North America and Europe. These trends underscore the need for targeted public health strategies to mitigate disease burden among older adults, given the growing contribution of this population to global IBD morbidity and disability.

Regarding mortality rates, recent U.S. analyses revealed stable all-cause mortality rates in IBD from 1999 to 2018, followed by an upturn between 2018 and 2020, with annual percent changes of +11.28 (CD) and +9.29 (UC). Mortality was higher in elderly and White adults, with crude mortality peaking in those aged ≥ 85 years (5.07 vs. 5.23 per 100,000 for CD and UC, respectively) [13]. The observed mortality increase between 2018 and 2020 coincides temporally with the COVID-19 pandemic.

The main epidemiological characteristics of Late-onset IBD are summarized in Table 1.

### 3.2. Pathophysiology and Pathogenesis of IBD in the Elderly

The pathophysiology of IBD in Late-onset IBD involves interplay between age-related immune changes, environmental exposures, comorbidities, and altered gut microbiota. While the fundamental immunopathogenesis of IBD remains a dysregulated immune response to intestinal microbiota in genetically susceptible hosts, aging introduces several modifiers that reshape disease behavior and outcomes.

Immunosenescence—the decline in immune competence with age—is a central contributor. Reduced T-cell function, altered cytokine signaling, and diminished immune specificity collectively foster a chronic low-grade proinflammatory state (“inflammaging”) characterized by elevated IL-6 and TNF-α levels, and reduced anti-inflammatory cytokines. This state predisposes older individuals to prolonged disease activity and attenuated healing responses.

Age-related microbiome alterations further exacerbate inflammation. Gut microbial diversity declines with age, with reductions in beneficial commensals (e.g., *Bifidobacterium* spp.) and increases in opportunistic species. These changes reduce short-chain fatty acid (SCFA) production, impair mucosal barrier integrity, and potentiate inflammatory cascades.

In Late-onset UC, recent evidence implicates a triad of gut barrier dysfunction—microbiota imbalance, epithelial barrier compromise, and defective immunoregulation—as key drivers of disease initiation during biological senescence [14].

Comorbidities (cardiovascular disease, diabetes, osteoporosis) and polypharmacy (notably NSAIDs and corticosteroids) contribute additional stressors to mucosal integrity and immune balance, often precipitating flares or complicating disease management. The cumulative effect is a unique disease phenotype—less extensive inflammation but greater systemic vulnerability—necessitating a nuanced therapeutic approach that accounts for the physiological context of aging.

### 3.3. Disease Phenotype in Late-Onset IBD

Late-onset IBD presents distinct phenotypic and clinical patterns compared with younger-onset disease, shaped mainly by immunosenescence, comorbidity burden, and age-related physiological changes. Disease manifestations are frequently more subtle, often resulting in delayed diagnosis and increased risk of complications.

#### 3.3.1. UC Phenotype

UC is the predominant form of IBD among older adults. Several consistent trends have emerged from cohort and registry data [15]:

- The disease is more often confined to the distal colon and less frequently extensive.
- The clinical course tends to be milder, with fewer acute severe flares, lower hospitalization rates, and lower corticosteroid requirements.
- Atypical and less severe presentations are common, manifesting as mild diarrhea, rectal bleeding, or nonspecific abdominal discomfort, which can mimic ischemic or diverticular colitis and delay diagnosis.

Multiple population-based studies have reported a tendency toward left-sided UC in Late-onset patients, though heterogeneity persists across geographic and ethnic cohorts [15]. Symptoms can overlap with colorectal malignancy, emphasizing the need for comprehensive evaluation, including endoscopic assessment and histologic confirmation.

#### 3.3.2. CD Phenotype

CD in the Late-onset patients displays several unique features [16,17]:

- Predominant colonic involvement rather than small bowel disease, in contrast with younger cohorts.
- A non-stricturing, non-penetrating inflammatory pattern is more common, leading to lower rates of fistula and abscess formation.
- Clinical presentation is often indolent—chronic diarrhea, mild abdominal discomfort, and unintentional weight loss—frequently mistaken for other gastrointestinal or metabolic disorders.

Earlier pre-biologic studies indicated that Late-onset patients with CD required fewer surgical interventions for perianal abscesses than younger adults [2]. A meta-analysis also identified age, gender, BMI, and low serum albumin as independent predictors of sarcopenia in Crohn’s disease [18], underscoring the broader systemic consequences of disease and inflammation in the aging population.

#### 3.3.3. Common Clinical Characteristics

Across both UC and CD, Late-onset patients often exhibit atypical or muted symptoms (e.g., mild diarrhea, anorexia, anemia, or nonspecific fatigue) that contribute to diagnostic delay. The differential diagnosis in this age group is wide, including ischemic colitis, diverticular disease, NSAID-induced enteropathy, and colorectal carcinoma.

Comorbidities such as cardiovascular disease, diabetes, and chronic pulmonary conditions complicate both presentation and management. Although disease activity may appear clinically milder, overall morbidity is greater, driven by frailty, malnutrition, sarcopenia, and increased infection susceptibility. Immunosuppressive therapy further elevates the risk of opportunistic infections and malignancy, while chronic corticosteroid use accelerates bone loss and fractures.

Frailty assessment and sarcopenia evaluation are increasingly recognized as essential prognostic tools in Late-onset IBD [19]. Fatigue and mood disturbances often accompany inflammatory activity, contributing to diminished quality of life, and should be actively managed. In a Swedish national cohort, Late-onset IBD was associated with a significantly increased risk of acute coronary syndrome, particularly within the first years after diagnosis [20].

A Danish population study compared patients diagnosed after age 70 with those diagnosed between 60 and 69 years, revealing significant differences in management and outcomes. Use of immunosuppressants and biologics was lower in the older subgroup, while rates of severe infections and postoperative complications were higher. No differences were noted in hospitalization, corticosteroid use, or disease incidence [21].

In summary, the unique pathophysiology of Late-onset IBD translates into a distinct clinical and therapeutic profile. Despite the perception of a milder inflammatory course, the coexistence of comorbidities, delayed diagnosis, and therapy-related adverse events often results in higher hospitalization and mortality rates. Current management strategies remain suboptimal in addressing the nuanced needs of elderly patients, underscoring the need for personalized, multidisciplinary care [22].

Table 2 indicates key differences between elderly-onset and young-onset IBD.

### 3.4. Diagnostic Challenges

Diagnosing Late-onset IBD is often complex due to atypical clinical presentations, overlapping comorbidities, and the effects of polypharmacy. These factors frequently lead to delayed or missed diagnoses, resulting in greater morbidity and suboptimal outcomes.

#### 3.4.1. Atypical and Nonspecific Presentations

Classic IBD symptoms—such as severe bloody diarrhea, abdominal pain, and weight loss—are often muted or absent in Late-onset patients. Instead, Late-onset adults may present with vague systemic manifestations, including fatigue, anorexia, anemia, low-grade fever, or unintentional weight loss. Such nonspecific findings are commonly attributed to other age-related disorders (e.g., ischemic colitis, diverticulitis, microscopic colitis, or colorectal cancer), thereby masking underlying IBD. Misattribution of symptoms to “normal aging” or other chronic conditions may postpone diagnosis for months or even years, leading to increased risk of complications such as intestinal strictures, perforations, and severe flares requiring hospitalization.

#### 3.4.2. Impact of Comorbidities and Polypharmacy

The coexistence of multiple chronic diseases (e.g., cardiovascular disease, diabetes mellitus, and chronic kidney disease) complicates both diagnosis and interpretation of gastrointestinal symptoms. Moreover, Late-onset adults frequently take several medications that may mimic or aggravate IBD symptoms. NSAIDs, widely used for musculoskeletal pain, can provoke mucosal injury and IBD flare-ups. Anticoagulants may exacerbate bleeding, while opioids and other analgesics alter bowel motility. These drug effects may confound diagnostic testing and clinical assessment, necessitating careful medication review during evaluation. Polypharmacy also reduces the reliability of specific laboratory or imaging findings. For instance, NSAID-induced enteropathy or ischemic colitis may produce endoscopic features that resemble IBD. Therefore, differential diagnosis requires comprehensive exclusion of medication-related gastrointestinal injury.

#### 3.4.3. Delayed Diagnosis and Diagnostic Accuracy

Delayed diagnosis in Late-onset patients has been associated with more advanced disease at presentation and poorer outcomes. In CD, diagnostic delays correlate with higher rates of stricturing and penetrating complications. Small-bowel CD is particularly challenging to identify due to its insidious onset and the limited accuracy of fecal calprotectin as a biomarker in older populations—its positive predictive value for small-bowel CD at levels > 200 μg/g is only 23.1% [23]. While calprotectin remains helpful in ruling out intestinal inflammation (high negative predictive value), it is insufficient for confirming small-bowel involvement in the Late-onset IBD. Therefore, clinicians must maintain a high index of suspicion and pursue further diagnostic modalities when appropriate.

#### 3.4.4. Recommended Diagnostic Approach

A structured, multimodal approach is essential for accurate diagnosis in this age group:

Endoscopy remains the gold standard for confirming IBD, allowing visualization and histologic evaluation of mucosal inflammation. Both colonoscopy and small-bowel enteroscopy (when indicated) should be performed, with particular attention to minimizing procedural risks in frail patients. Cross-sectional imaging such as computed tomography (CT) or magnetic resonance (MR) enterography can delineate disease extent, identify complications, and distinguish IBD from malignancy or ischemic lesions. Capsule endoscopy may be reserved for cases where small-bowel involvement is suspected and other modalities are inconclusive. Biomarker assessment, including fecal calprotectin and serum inflammatory markers (CRP, ESR), helps detect active inflammation and monitor response to therapy. However, interpretation must consider age-related changes in baseline inflammatory activity.

When evaluating Late-onset patients, clinicians should maintain a broad differential diagnosis that includes ischemic, infectious, and microscopic colitis, as well as medication-related enteropathies. Polypharmacy, comorbid conditions, and frailty should always inform the diagnostic plan.

**Figure 3:**
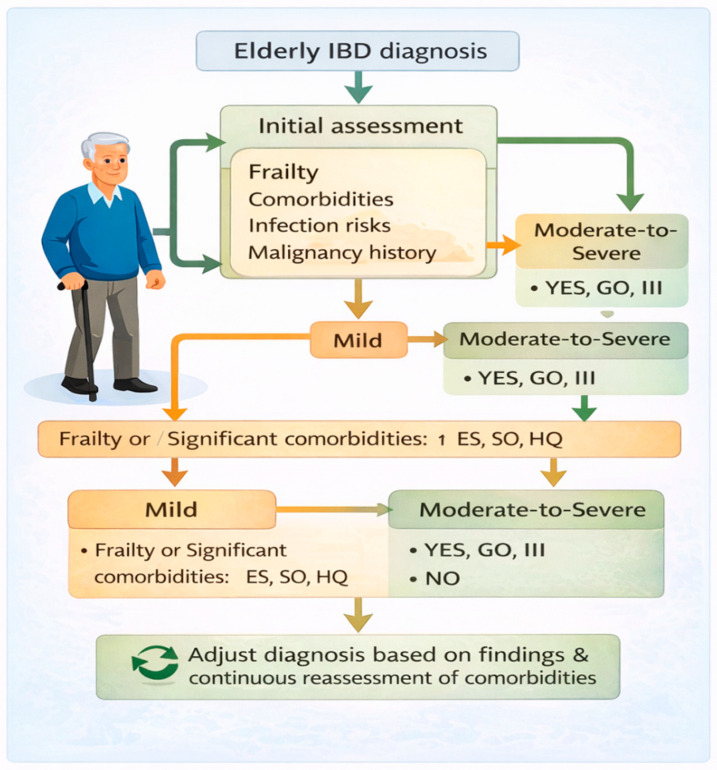
Diagnostic approach in elderly people with IBD.

Figure 3 shows the diagnostic approach applied in elderly IBD patients.

#### 3.4.5. Pre-Treatment Screening and Baseline Assessment

Before initiating immunosuppressive or biologic therapy, a thorough baseline evaluation is required:

- Screening for latent infections (tuberculosis, hepatitis B, and C).
- Vaccination review (influenza, pneumococcus, shingles).
- Nutritional assessment (malnutrition and vitamin D deficiency are standard in older IBD patients).
- Geriatric and frailty assessment to estimate procedural risk and therapeutic tolerance.

This structured, individualized diagnostic and baseline approach ensures early, accurate diagnosis while minimizing iatrogenic complications and inappropriate treatments [24,25,26,27,28] (Table 3).

### 3.5. Therapeutic Principles and Strategies in Late-Onset Patients with IBD

The management of Late-onset IBD presents unique challenges, primarily due to the interplay between frailty, multimorbidity, and polypharmacy. Although the therapeutic goals—achieving remission, preventing relapse, and maintaining quality of life—are identical to those in younger patients, treatment in the elderly requires greater emphasis on safety, tolerability, and individualized decision-making.

A patient-centered approach should balance efficacy with the potential risks of immunosuppression, infection, malignancy, and drug interactions. The general principle is to begin with the least systemically immunosuppressive effective therapy, while continuously reassessing frailty, comorbidities, and functional status. Close collaboration with geriatric specialists, pharmacists, and nutritionists is often necessary.

Supportive measures—nutritional optimization, osteoporosis prevention, immunization, and fall risk assessment—are integral to comprehensive care [29]. When surgery is indicated, preoperative optimization and minimally invasive techniques can reduce morbidity and mortality, which are higher in this population. Unfortunately, misconceptions regarding the indications and risks of modern IBD therapies often lead to under-treatment and overreliance on corticosteroids in older adults.

#### 3.5.1. Conventional Therapies

##### Mesalamine

Mesalamine remains a cornerstone for mild-to-moderate UC due to its favorable safety profile. It is generally well-tolerated and effective for both induction and maintenance of remission. However, renal toxicity is a concern in elderly patients, who often have reduced glomerular filtration rates. Regular monitoring of serum creatinine is recommended during therapy. Other rare adverse events include diarrhea, allergic reactions, and interstitial nephritis. Overall, mesalamine remains the safest first-line option for Late-onset adults with mild UC.

##### Corticosteroids

Corticosteroids (prednisone, prednisolone, methylprednisolone, budesonide) are effective for inducing remission during acute flares but should be used only for short-term treatment in elderly patients. Systemic corticosteroids are associated with numerous adverse effects—including infection, osteoporosis, cataracts, hypertension, and hyperglycemia—that are magnified with age. Budesonide, a locally acting corticosteroid with minimal systemic exposure, is preferable for localized CD or UC. Patients exposed to prednisone report more adverse events and higher rates of mood disturbances than those receiving budesonide [30]. Rapid transition to steroid-sparing maintenance therapy is critical.

##### Immunomodulators (Thiopurines)

Thiopurines (azathioprine and 6-mercaptopurine) are used for maintenance of remission but require particular caution in older adults. The risk of myelosuppression, infection, and hepatotoxicity increases with age, and drug–drug interactions are common due to polypharmacy. Regular blood monitoring is mandatory. Long-term thiopurine use is linked to increased malignancy risk, especially nonmelanoma skin cancer and lymphoproliferative disorders; thus, its use should be restricted to carefully selected cases. Overall, thiopurines are less favored in Late-onset patients, where safer alternatives are now available.

Table 4 summarizes the advantages and special considerations of conventional therapies in elderly patients with IBD.

##### Biological Therapies: Evidence and Practical Guidance

The advent of biologics has revolutionized IBD management, including in older populations. However, most pivotal trials underrepresent elderly patients, and real-world safety data are essential for guiding treatment selection.

#### 3.5.2. Anti-TNF Agents

##### Efficacy

Anti-TNF agents (infliximab, adalimumab, certolizumab pegol (in the USA), golimumab) remain the most extensively studied biologics in Late-onset IBD. They are highly effective for induction and maintenance of remission in both UC and CD [31,32,33,34,35].

##### Safety

Late-onset adults experience similar relative efficacy but a higher absolute risk of serious infections and hospitalizations. Combination therapy with thiopurines confers additional immunosuppressive burden and is generally discouraged. Anti-TNF therapy should be avoided in patients with moderate to severe heart failure due to exacerbation risk.

##### Practical Recommendations

- Use as monotherapy when rapid disease control is required.
- Perform thorough infection screening and vaccination before initiation.
- Employ therapeutic drug monitoring (TDM) to optimize dosing and minimize toxicity.

#### 3.5.3. Anti-Integrin Agents (Vedolizumab)

##### Mechanism and Efficacy

Vedolizumab, a gut-selective anti-α4β7 integrin antibody, has demonstrated efficacy in inducing and maintaining remission in both UC and CD (GEMINI program) [36,37].

##### Safety in the Late-Onset IBD

Due to its localized intestinal activity, vedolizumab has a favorable safety profile, with lower systemic infection rates than anti-TNF agents. In real-world studies, Late-onset patients achieved remission rates similar to those of younger cohorts, albeit with a slower onset of action [38,39].

##### Practical Recommendations

Vedolizumab is considered a first-line biologic option for Late-onset patients at high infection risk or with significant comorbidities. Combination with a short corticosteroid taper is reasonable to accelerate early disease control.

#### 3.5.4. Anti-IL-12/23 Agents (Ustekinumab)

##### Efficacy and Safety

Ustekinumab has proven efficacy in moderate-to-severe CD and UC (UNITI/IM-UNITI trials) [40,41,42]. Registry data suggest that it maintains a strong safety profile with low infection rates in Late-onset adults. Comparative analyses show remission and infection rates similar to those with anti-TNF agents [43].

##### Practical Recommendations

Ustekinumab represents an excellent choice for Late-onset patients with systemic disease requiring a well-tolerated biologic, particularly after anti-TNF or vedolizumab failure.

##### Emerging Biologics

New biologics targeting alternative pathways are under investigation:

- Etrasimod (S1P receptor modulator)—oral, effective in UC [44].
- Obefazimod (miR-124 enhancer)—favorable long-term efficacy in UC [45].
- Mirikizumab and Guselkumab (anti-IL-23 antibodies)—positive phase 3 data for UC and CD [46,47,48].
- Risankizumab—effective and well-tolerated in both naïve and biologic-exposed CD patients [49].

While promising, long-term safety data in Late-onset populations remain limited.

##### Small-Molecule Therapies (JAK Inhibitors)

JAK inhibitors (tofacitinib, upadacitinib, filgotinib) offer potent oral alternatives for moderate-to-severe UC and CD [50,51]. They have a rapid onset and no immunogenicity but carry significant safety concerns—thromboembolic events, herpes zoster, dyslipidemia, and cardiovascular risk—particularly relevant in Late-onset patients [52,53,54]. Comparative studies indicate that upadacitinib achieves higher remission rates than tofacitinib or filgotinib but with increased acne and lipid alterations [55]. Real-world data support their long-term efficacy, yet JAK inhibitors should be reserved for patients in whom the benefits clearly outweigh cardiovascular risks [56,57,58].

**Figure 4:**
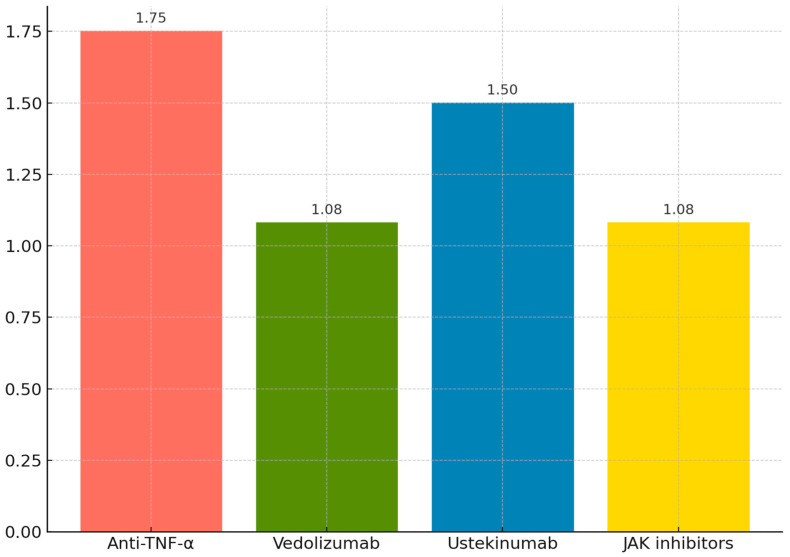
Relative risk of serious infection among patients aged ≥60 years compared to younger cohorts receiving biologic therapy. Data synthesized from randomized clinical trials and real-world registry studies [50,59,60,61,62,63,64,65].

Figure 4 shows the risk of infection in IBD patients aged 60 or older versus those aged <60 by biologic class. As indicated in this Figure, anti-TNF therapy in Late-onset patients was associated with an increased risk of serious infections compared to younger cohorts (OR: 1.45; 95% CI: 1.10–1.91. However, as this study is a structured narrative synthesis rather than a formal meta-analysis (due to significant heterogeneity in study designs), quantitative pooling of data was not feasible. Compared to vedolizumab and JAK inhibitors, anti-TNF-α agents and ustekinumab have higher relative infection risks than, which show lower relative infection risks in Late-onset populations.

Table 5 summarizes the typical adult dosing, efficacy and Safety of the main Biologic Agents used in Elderly IBD.

##### Surgical Outcome

The surgical treatment of Late-onset IBD patients is beyond the scope of this systematic review. In general, surgery may be necessary, but elderly patients have elevated perioperative morbidity and mortality rates. Preoperative optimization, minimally invasive techniques, and geriatric support improve outcomes [66]. Segmental colectomy may be performed in selected patients with IBD and colorectal cancer, such as Late-onset patients with a short duration of disease, patients with mild colitis, especially if diagnosed preoperatively, in patients with high surgical risk and severe comorbidities, as well as in patients whose colorectal cancer is sporadic and not related to UC. However, patients who have undergone segmental colectomy should be monitored postoperatively for the development of dysplasia and/or cancer recurrence [67]. In a study published before the era of biologic agents, the authors noted that significantly fewer elderly patients with UC underwent surgery for their disease than younger patients (6.25% vs. 22.3%; *p* = 0.027) [3]. Early surgeries in CD were higher in Late-onset patients with IBD [2]. A recent study compared the results of surgical procedures performed on patients with IBD under the age of 60 and patients over the age of 60. The adjusted Hazard Ratios for a new surgery among the elderly with UC and CD were 0.69 and 0.98, respectively. In the Late-onset UC, the adjusted Hazard Ratios of infections within 6 and 12 months after surgery were 1.07 and 0.85, respectively, while in the Late-onset group the corresponding features were 1.45 and 1.26, respectively, which suggests that elderly patients with CD, but not with UC, had an increased risk of serious infections within 6 months of surgery and that Late-onset patients with IBD did not have an increased risk of new abdominal surgery within two years of the first surgery [68].

### 3.6. Special Considerations in the Management of Late-Onset Patients with IBD

The management of IBD in Late-onset adults requires an integrated, multidisciplinary approach that extends beyond pharmacologic treatment. Aging introduces several additional considerations—immunization, malignancy surveillance, polypharmacy, frailty, and psychosocial factors—that profoundly influence therapeutic safety and outcomes. Optimizing these domains is critical for improving quality of life and minimizing treatment-related harm.

#### 3.6.1. Vaccination and Infection Prevention

Before initiating immunosuppressive or biologic therapy, Late-onset patients should undergo a comprehensive vaccination review. Age-related immunosenescence reduces vaccine efficacy, yet vaccination remains the most effective preventive strategy against infectious complications.

Recommended vaccinations include influenza, pneumococcal (PCV20 or sequential PCV15–PPSV23), hepatitis B, and recombinant zoster vaccine (preferred over live zoster) [52,53]. Live vaccines are contraindicated in patients receiving or scheduled to receive significant immunosuppression. Vaccination ideally precedes biologic initiation by at least 2–4 weeks. An annual review of vaccination status is recommended, especially in patients on polypharmacy or those who are immunocompromised. Finally, proactive infection prevention, including screening for latent tuberculosis, hepatitis B and C, and HIV, remains standard practice before starting immunomodulators or biologics.

For Late-onset patients with IBD, COVID-19 vaccination is considered both safe and essential, as advanced age remains the primary risk factor for severe outcomes. Clinical data indicate that these patients do not experience a higher rate of disease flares following vaccination compared to the general population; in fact, Late-onset patients often report fewer systemic side effects (such as fever or fatigue) than younger cohorts. While the majority of IBD patients mount a robust immune response, immunogenicity can be blunted in those receiving certain therapies, particularly high-dose corticosteroids or anti-TNF agents (especially when used in combination with immunomodulators). However, cell-mediated immunity often remains preserved even when antibody titers are lower. Consequently, 2025–2026 guidelines from the British Society of Gastroenterology and the American College of Gastroenterology strongly recommend that all Late-onset IBD patients remain “up to date” with the latest seasonal vaccine formulations. For those over age 65 or those on advanced immunosuppressive therapies, current recommendations often advise a second dose of the updated seasonal vaccine or a 6-monthly booster schedule to account for waning immunity and the emergence of new variants [53].

#### 3.6.2. Cancer and Malignancy Risk

Cumulative immunosuppression and age-related genomic instability contribute to increased malignancy risk in Late-onset IBD patients. Long-term thiopurine therapy is associated with higher rates of nonmelanoma skin cancer and lymphoproliferative disorders. In general:

- Biologic monotherapy (vedolizumab, ustekinumab) appears to have a more favorable long-term malignancy profile, though continued surveillance is warranted.
- Age-appropriate cancer screening—including colonoscopy, dermatologic evaluation, and gender-specific screening (breast, prostate, cervical)—is essential.
- Oncologic vigilance should continue even after discontinuation of immunosuppressive therapy.

#### 3.6.3. Polypharmacy and Drug Interactions

Polypharmacy is highly prevalent in Late-onset patients with IBD and strongly correlates with adverse outcomes, hospitalizations, and reduced quality of life. Kochar et al. [69] reported that 86% of older adults with IBD used ≥5 concomitant medications, and 45% exhibited severe polypharmacy (≥10 medications), which was associated with poorer functional status and life quality. In general:

- Drug–drug interactions can alter IBD medication efficacy or increase toxicity.
- NSAIDs and corticosteroids may exacerbate mucosal injury or bleeding.
- Anticoagulants and antiplatelet agents can increase the risk of gastrointestinal bleeding.
- Opioids impair bowel motility and mask disease activity.
- Antibiotics and proton pump inhibitors disrupt gut microbiota, potentially aggravating inflammation.
- Deprescription strategies, coordinated with primary care and pharmacy teams, should be implemented regularly to minimize unnecessary medication burden [70].

#### 3.6.4. Frailty and Functional Assessment

Frailty—a multidimensional syndrome reflecting reduced physiologic reserve—is an independent predictor of adverse outcomes in Late-onset IBD patients. Frail patients experience higher rates of hospitalization, infections, and postoperative complications, and derive less benefit from aggressive immunosuppression. A structured frailty assessment (e.g., Clinical Frailty Scale, gait speed, grip strength) should guide therapeutic intensity and shared decision-making. Integrating geriatric assessment tools allows clinicians to individualize care, balance efficacy with safety, and align treatment goals with patient preferences.

#### 3.6.5. Supportive and Nutritional Measures

Comprehensive IBD care in Late-onset extends beyond pharmacologic management and includes:

- Routine nutritional screening for malnutrition, vitamin D deficiency, and anemia.
- Optimization of bone health, including calcium and vitamin D supplementation and bone density monitoring.
- Vaccination maintenance as discussed above.
- Fall risk assessment and physical activity promotion to counteract sarcopenia and frailty.

Such measures improve resilience and treatment tolerance while reducing the risk of hospitalization.

#### 3.6.6. Psychosocial and Cognitive Factors

Psychosocial well-being is often underrecognized in Late-onset IBD care. Depression, anxiety, social isolation, and financial constraints may impair treatment adherence and outcomes. Cognitive decline and reduced health literacy further complicate therapy management. Regular psychosocial assessment, caregiver engagement, and integration of mental health and social work support are essential for holistic care.

In summary, Late-onset patients with IBD constitute a vulnerable population requiring nuanced management strategies that integrate immunization, malignancy prevention, rational pharmacotherapy, and functional support. A proactive, multidisciplinary, and individualized approach—anchored in shared decision-making—optimizes both safety and quality-of-life outcomes in this rapidly expanding patient group.

**Scheme 1:**
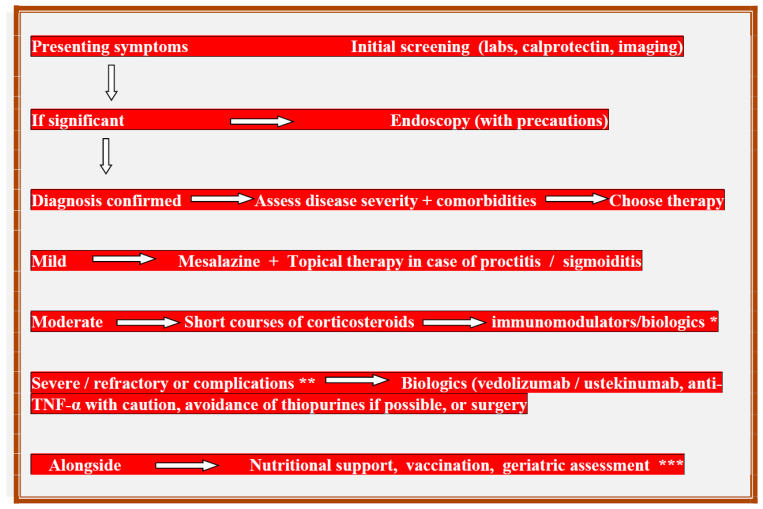
Management Approach in a Late-onset Patient with Suspected IBD. (*): vedolizumab or ustekinumab monotherapy (**): choose another class (e.g., ustekinumab after anti-TNF, vedolizumab, or JAK inhibitors only when benefits outweigh the cardiovascular and thrombotic risk. (***): Bone health optimization, medication reconciliation, and monitoring.

Scheme 1 summarizes the management approaches in an elderly patient with suspected IBD.

## 4. Discussion

The present systematic review consolidates and critically analyzes the epidemiological, clinical, and therapeutic characteristics of IBD in the Late-onset population, a subgroup of growing global relevance. With increasing life expectancy and a rising number of incident IBD cases in older adults, the intersection between aging and chronic intestinal inflammation presents unique challenges for clinicians and public health systems. Our findings support the notion that Late-onset IBD, though accounting for approximately 10–15% of new diagnoses, represents a distinct clinical and biological entity that warrants tailored diagnostic and management strategies.

Regarding the epidemiology of Late-onset IBD, the available data across the past three decades consistently demonstrate that the global burden of IBD among individuals aged ≥60 years is increasing. Although age-standardized incidence rates appear stable or declining in high-income regions, absolute numbers continue to rise due to population aging. Studies from the Global Burden of Disease program [7,8,9] confirm that by 2030, one-third of all IBD patients may be over 60 years of age. This demographic shift demands greater focus on age-appropriate disease surveillance, vaccination policies, and therapeutic risk stratification. Interestingly, no consistent sex differences were observed in global incidence, although regional variations—such as higher incidence among elderly females in the United States—have emerged. These trends underline the importance of incorporating age as an essential variable in future IBD epidemiology. This epidemiological distribution of Late-onset IBD provides a clear rationale for individualized therapeutic approaches, as Late-onset patients exhibit heterogeneous risk profiles related to immunosenescence, comorbidities, and treatment-related adverse events.

The pathogenesis of IBD in Late-onset IBD shares core immunological mechanisms with younger-onset disease but is profoundly influenced by immunosenescence and inflammaging. Age-related decline in adaptive immunity, coupled with heightened systemic inflammatory tone, reshapes the immune response to intestinal microbiota. Reduced T-cell diversity and increased levels of proinflammatory cytokines (IL-6, TNF-α) create a milieu that favors persistent low-grade inflammation. Simultaneously, gut microbial diversity decreases with age, leading to reduced production of short-chain fatty acids, which are essential for mucosal health. These changes not only affect disease behavior but also modulate therapeutic response, particularly to immunomodulators and biologics. Hence, elderly IBD may represent a model of chronic inflammation superimposed upon systemic frailty rather than a simple extension of the same disease seen in younger individuals.

Concerning the clinical phenotype and the “nuances” of clinical behavior in this population, our synthesis corroborates that UC predominates over CD among Late-onset cases, often presenting with left-sided or distal colitis. At the same time, CD more frequently involves the colon and manifests a non-stricturing, non-penetrating pattern. Atypical or muted symptoms—such as mild diarrhea, anemia, or weight loss—often delay diagnosis. Furthermore, differential diagnoses in the Late-onset IBD are broader, encompassing ischemic, infectious, and drug-induced colitides. This diagnostic ambiguity contributes to under-recognition and suboptimal early management. Utilization of fecal calprotectin, although valuable for ruling out inflammation, may have limited specificity in this age group. Therefore, comprehensive endoscopic and imaging assessment remains indispensable.

Treatment of Late-onset IBD must balance efficacy and safety, considering comorbidities, polypharmacy, and frailty. Mesalamine remains first-line therapy for mild to moderate UC due to its favorable safety profile. Corticosteroids, while effective for induction, should be minimized due to well-documented risks of osteoporosis, metabolic complications, and infection. The overuse of corticosteroids in older adults—stemming from fear of biologic therapy—is a recurrent clinical pitfall. Thiopurines should be used with caution, given the increased risks of myelotoxicity and malignancy in this age group.

Current data suggest prioritizing biologic monotherapy over combination regimens whenever possible to reduce infection risk. Among biologic classes, vedolizumab and ustekinumab have shown the most favorable balance between efficacy and safety in Late-onset IBD populations. Real-world registry data consistently report lower systemic infection rates compared with anti-TNF agents. Anti-TNF therapies, though effective, are associated with higher rates of serious infections and hospitalizations, especially when combined with thiopurines. JAK inhibitors represent a potent oral alternative, but their use should be restricted to patients without significant cardiovascular or thromboembolic risk.

Collectively, the therapeutic approach should focus on minimizing systemic immunosuppression, individualized frailty assessment before biologic initiation, vaccination and infection screening, and shared decision-making integrating patient values and functional status.

Vaccination against SARS-CoV-2 is strongly recommended in Late-onset patients with inflammatory bowel disease, including those receiving biologic or small-molecule therapies. Available real-world data and international guidelines indicate an acceptable safety profile and adequate immunogenicity in this population, while the benefits of vaccination clearly outweigh potential risks.

Other factors influence the outcome of treatment. Polypharmacy is nearly universal among Late-onset IBD patients, with >80% using ≥5 concurrent medications [53]. Drug–drug interactions, especially involving NSAIDs, anticoagulants, and corticosteroids, compound risks of bleeding, infection, and metabolic toxicity. Frailty, cognitive impairment, and nutritional deficits also influence adherence and outcomes. Integrating frailty assessment tools (e.g., Clinical Frailty Scale) and comprehensive geriatric evaluation can guide the intensity of immunosuppressive therapy and surgical decision-making. These non-gastrointestinal dimensions of care are critical to optimize both survival and quality of life.

Surgery in the Late-onset patients with IBD requires special attention. Generally, surgical rates among Late-onset IBD patients are usually lower for UC and similar or slightly higher for CD compared to younger cohorts. However, postoperative morbidity and infection rates are significantly higher. Minimally invasive techniques, preoperative optimization, and postoperative surveillance for dysplasia are key strategies. Multidisciplinary perioperative care—including nutritional, cardiac, and anesthetic evaluation—is particularly beneficial in this age group.

Finally, the steady increase in Late-onset IBD cases signifies a need for public health frameworks addressing prevention, early diagnosis, and specialized management. Educational programs targeting general practitioners and internists could reduce diagnostic delays. Additionally, most clinical trials systematically exclude older adults, resulting in a paucity of high-quality evidence. Future research should focus on age-stratified safety and pharmacokinetic studies of biologics and small molecules, mechanisms of immunosenescence and alterations in the microbiome, and the cost-effectiveness of geriatric-inclusive care pathways.

Nevertheless, there are some limitations regarding the current evidence. In the authors’ opinion, the main restriction in interpreting available data lies in the heterogeneity of definitions of Late-onset IBD, variable study designs, and the underrepresentation of older adults in randomized controlled trials. Observational and registry studies, while valuable, are prone to confounding. Moreover, most datasets originate from Western populations, limiting generalizability to low- and middle-income regions. Despite these constraints, converging trends strongly validate the distinctiveness of elderly IBD as a clinical entity. Regarding the data presented in this review, publication bias cannot be excluded, as studies reporting neutral or negative outcomes may be underrepresented. The overall certainty of evidence was considered low to moderate, primarily due to study heterogeneity and the limited availability of randomized data in elderly populations. Regarding the certainty of evidence for key outcomes (e.g., safety of biologics) based on GRADE principles and framework, the overall certainty of evidence for the efficacy of advanced therapies in the elderly was “moderate to high”, primarily due to the observational nature of most studies. This was consistent across multiple large-scale registries and clinical trials. A number of other limitations such as the lack of RCTs specifically for the elderly population were important barriers.

## 5. Conclusions

Late-onset IBD represents a unique and increasingly prevalent clinical subset, shaped by the biological processes of aging, comorbidity, and frailty. Although the fundamental mechanisms mirror those of younger-onset disease, the clinical manifestations, diagnostic challenges, and therapeutic risks differ substantially. A patient-centered, geriatric-informed approach—emphasizing infection prevention, vaccination, frailty assessment, and safe biologic use—is essential for optimal outcomes. Epidemiological data in Late-onset patients further support the necessity of personalized, risk-adapted treatment strategies rather than uniform therapeutic algorithms.

Future strategies should focus on incorporating elderly-specific endpoints into clinical trials, enhancing cross-disciplinary collaboration between gastroenterologists and geriatricians, and developing precision medicine algorithms that balance efficacy with safety. As the IBD population ages, these measures will be pivotal to transforming care delivery from disease-centered to patient-centered longevity medicine.

## Figures and Tables

**Table 1:** Key epidemiological features of elderly-onset IBD.

Feature	Ulcerative Colitis	Crohn’s Disease
Proportion of elderly-onset cases	~60%	~40%
Male-to-female ratio	Equal or slight male predominance	Slight female predominance
Typical age at onset	60–75 years	60–70 years

**Table 2:** Key Differences between Elderly-Onset and Young-Onset IBD.

Characteristic	Elderly-Onset IBD	Young-Onset IBD
Disease location	Distal or left-sided colitis; predominantly colonic CD	Extensive colitis; ileal or leocolonic CD
Disease course	Often milder, fewer complications	Typically more aggressive
Comorbidities	Common; influence treatment choice	Less frequent
Treatment challenges	Polypharmacy, adverse events, limited clinical trial data	Longer cumulative exposure to immunotherapy

**Table 3:** Pre-Biologic Evaluation in Late-onset Patients with IBD.

Evaluation Domain	Key Elements
History	Tuberculosis exposure, prior malignancy, vaccination and cardiac history
Laboratory and Imaging Tests	TB IGRA or PPD; chest X-ray if indicated; HBsAg, anti-HBc, HCV serology; CBC, renal and hepatic function, CRP, fecal calprotectin
Vaccinations	Influenza, pneumococcal, recombinant zoster, hepatitis B (if non-immune)
Medication Review	Corticosteroids, anticoagulants, immunosuppressants
Functional Assessment	Frailty, cognition, social support and care needs

TB: Tuberculosis; IGRA: interferon gamma release assay; PPD: purified protein derivative; HBsAg: Hepatitis B surface antigen; HBc: capsid protein of hepatitis B virus; HCV: complete blood count; CBC: complete blood count; CRP: C-reactive protein.

**Table 4:** Conventional Therapies in Late-onset IBD Patients.

Therapy	Advantages	Risks and Special Considerations
Mesalamine	Safe, well-tolerated, effective for mild UC	Renal toxicity (monitor), limited effect in severe disease
Corticosteroids	Rapid symptom relief	Osteoporosis, infection, hypertension, hyperglycemia
Thiopurines	Steroid-sparing	Myelosuppression, liver toxicity, malignancy risk, drug interactions

**Table 5:** Comparative Efficacy and Safety of Biologic Agents in Late-onset IBD.

Agent/Class	Typical Adult Dosing	Relative Efficacy (Moderate–Severe)	Safety Considerations in Late-Onset IBD
Anti-TNF (Infliximab, Adalimumab)	Standard induction/maintenance	High efficacy; rapid onset	Higher systemic infection risk; avoid combination with thiopurines
Vedolizumab	300 mg IV at weeks 0, 2, 6 → every 8 weeks	Strong efficacy; slower onset	Gut-selective; low systemic infection rate
Ustekinumab	Weight-based IV induction → 90 mg SC q8–12 weeks	Robust efficacy, especially post–anti-TNF	Favorable infection and malignancy profile
JAK inhibitors	Oral fixed dosing	High efficacy, rapid onset	Thrombosis and cardiovascular risk; caution in elderly

IBD: Inflammatory Bowel Disease; TNF: Tumor Necrosis Factor-α; SC: subcutaneus; JAK: Janus kinases.

## Data Availability

The original contributions presented in this study are included in the article and Supplementary Materials. Further inquiries can be directed to the corresponding author.

## Supplementary Materials

The following supporting information can be downloaded at https://www.mdpi.com/article/10.3390/jpm16020059/s1: Table S1: Main findings of the studies used in this systematic review. Table S2: PRISMA checklist.
