# Supplementary material for: Challenges and Treatment Strategies in Elderly Patients with Inflammatory Bowel Disease: A Systematic Review and Narrative Synthesis
Source: J Pers Med. 2026 Jan 23;16(2):59. doi: 10.3390/jpm16020059 (PMC12941479; doi:10.3390/jpm16020059)
Supplement: Supplementary file 1 [file jpm-16-00059-s001.zip › Table S1.pdf]

Search Strategy,
("Inflammatory Bowel Diseases"[Mesh] OR "ulcerative colitis"[Title/Abstract] OR "Crohn Disease"[Title/Abstract] OR "IBD"[Title/Abstract] OR "inflammatory bowel disease"[Title/Abstract])
AND
("Aged"[Mesh] OR elderly [Title/Abstract] OR "older adult"[Title/Abstract] OR "late-onset"[Title/Abstract] OR "late onset"[Title/Abstract] OR "older patients"[Title/Abstract])
AND
("biologic"[Title/Abstract] OR biologic*[Title/Abstract] OR "vedolizumab"[Title/Abstract] OR "ustekinumab"[Title/Abstract] OR "infliximab"[Title/Abstract] OR "adalimumab"[Title/Abstract] OR "anti-TNF"[Title/Abstract] OR "tofacitinib"[Title/Abstract] OR "upadacitinib"[Title/Abstract] OR "JAK inhibitor"[Title/Abstract] OR "janus kinase"[Title/Abstract])
AND
("safety"[Title/Abstract] OR "infection"[Title/Abstract] OR "adverse event"[Title/Abstract] OR "efficacy"[Title/Abstract] OR "effectiveness"[Title/Abstract] OR "dose"[Title/Abstract] OR "dosing"[Title/Abstract] OR "outcomes"[Title/Abstract])

Filters: Humans; English; Publication date from 2000/01/01 to 2025/01/01.

Table S1. Type and main results of the studies used in this systematic review.

Reference	Type of Study	Topic	Main Findings
J Crohns Colitis 2025 [2]	Prospective population-based cohort of IBD patients	Epidemiological and clinical features in Elderly vs Adult onset IBD patients	Elderly-onset IBD represents an increasing proportion of IBD patients, with high exposure to biologicals in the UC patients. Surgery rates were similar in Elderly vs Adult Onset.
United European Gastroenterol J 2025 [5]	Comparative study	Comparison of disease phenotypes and treatment exposures between adult and elderly-onset IBD patients	Elderly-onset IBD shows higher prevalence of colon-only IBD. Treatment in elderly-onset IBD favors 5-ASA. It also shows reduced biological use, with preferences for Vedolizumab.
Lancet 2017 [6]	Systematic review	Assessing the incidence and prevalence of IBD around the world	Although the incidence of IBD is stabilising in western countries, burden remains high, as prevalence surpasses 0·3%.
Front Aging 2024 [8]	Epidemio-logy	Description of the historical trends of IBD burden in the elderly from 1990 to 2021 and forecast trends up to 2051	The absolute burden of IBD in the elderly population is going to increase substantially by 2051, despite decreasing age-standardized rates.
Curr Opin Gastroenterol 2025 [12]	Review	Approaches to managing older adults with small bowel CD.	Management of Elderly-onset IBD should be tailored to patient preference and frailty.
Am J Med Sci. 2025 [18]	Systematic review	Investigation of factors influencing sarcopenia in CD.	The occurrence of sarcopenia is mainly influenced by gender, BMI, age, and low albumin levels.

Clin Gastroenterol Hepatol 2025 [21]	Epidemio-logy	Denmark Nationwide Population-Based Cohort Study in Elderly and very Elderly –onset IBD	Patients diagnosed with very late-onset IBD have a higher relative burden of disease- and aging-related complications, with limited use of steroid-sparing strategies and surgery, compared with late-onset IBD
J Crohns Colitis. 2025 [24]	Practical guideline	Diagnosis and treatment of IBD	A comprehensive and practical guidance on the diagnostic and monitoring of patients with IBD
J Crohns Colitis. 2025 [25]	Practice guideline	Management of IBD	There are significant challenges to adopting a holistic management strategy in Low and Middle Income Countries.
Gastroenterology. 2021 [26]	Practice guideline	Vaccination strategies (Live vaccines)	Appropriate vaccination is critical to optimize patient's outcomes. Live vaccines are recommended in patients not on immunosuppressives, but not for those using immunosuppressives.
Gastroenterology 2021 [27]	Practice guideline	Vaccination strategies (Inactivated vaccines)	IBD is not a contraindication to the use of inactivated vaccines, but immunosuppressive therapy may reduce vaccine responses
Dig Liver Dis. 2023 [29]	Review	Treatment strategies in the elderly	Principles of management of IBD in the elderly
J Crohns Colitis. 2025 [30]	Clinical study	Corticosteroids in IBD	Adverse events associated with IBD-related corticosteroid use are frequent. AEs affect patients' quality of life
Curr Gastroenterol Rep 2025 [31]	Review	Management of IBD in the elderly	The majority of literature revolves around retrospective and observational studies. Multidisciplinary care models must be applied in elderly IBD patients
Aliment Pharmacol Ther 2015 [32]	Clinical study	Treatment of CD	Combination therapy is more effective in achieving remission vs. azathioprine or infliximab monotherapy
N Engl J Med. 2004 [33]	Clinical study	Treatment of CD	Continuous Infliximab treatment in patients with fistulizing CD who responded to induction therapy with infliximab have an increased likelihood of a sustained response on infliximab if it is continued every 8 weeks
Lancet. 2002 [34]	Clinical trial	Treatment of CD with biologics	Infliximab treatment should be maintained indefinitely every 8 weeks
N Engl J Med 2005 [35]	Randomized clinical trial	Treatment of UC	Patients with moderate-to-severe active UC treated with infliximab as a maintenance treatment have better clinical response at weeks 8, 30, and 54 than those receiving placebo.
N Engl J Med. 2013 [36]	Clinical trial	Treatment of UC with biologics	Vedolizumab was more effective than placebo as induction and maintenance therapy for UC.
N Engl J Med. 2019 [37]	Clinical trial	Vedolizumab versus Adalimumab	Vedolizumab was superior to adalimumab with respect to

			achievement of clinical remission and endoscopic improvement in acive UC.
J Clin Med. 2024 [38]	Multicentric retrospective study	Ustekinumb versus vedolizumab in IBD	Ustekinumab and vedolizumab show comparable effectiveness and safety in the elderly IBD population
J Clin Gastroenterol. 2024 [39]	Metaanalysis	Vedolizumab in elderly vs gunger patients with IBD	Vedolizumab is equally safe and effective for clinical and endoscopic remission in elderly and younger populations
J Crohns Colitis. 2020 [40]	Randomized controlled trial	Ustekinumab in IBD	Continued treatment with subcutaneous ustekinumab maintained clinical response and remission through 3 years in patients who responded to induction therapy.
N Engl J Med. 2012 [41]	Clinical trial	Ustekinumab in CD	Patients with moderate-to-severe CD resistant to anti-TNF, had an increased rate of response to induction with ustekinumab.
N Engl J Med. 2016 [42]	Clinical trial	Ustekinumab in CD	Patients with CD who were treated with IV ustekinumab had a higher rate of response compared to placebo. Ustekinumab maintained remission.
Inflamm Bowel Dis. 2025 [43]	Comparative study	Safety and effectiveness of ustekinumab versus anti-TNF	A comparable safety and effectiveness for ustekinumab and anti-TNF agents in elderly CD patients was seen.
N Engl J Med. 2017 [50]	Clinical trial	Tofacitinib in UC	In patients with active UC, tofacitinib was more effective as induction and maintenance therapy than placebo.
Lancet Gastroenterol Hepatol 2025 [51]	Clinical trial, Interim analysis	Upadacitinib in UC	There was positive long-term risk-benefit profile for upadacitinib 15 mg and 30 mg among patients with active UC.
Gut 2019 [53]	Review	Treatment of IBD	Guidelines in the management of IBD
Gastroenterology 2019 [54]	Review	Management of UC	Treatment guidelines in UC
Aliment Pharmacol Ther 2025 [55]	Comparative study	Upadacitinibe in refractory UC	Filgotinib or tofacitinib may be considered as an upfront JAK inhibitor before using upadacitinib
Aliment Pharmacol Ther 2025 [56]	Multicenter (real-world) study	Upadacitinib	In highly refractory CD patients, upadacitinib resulted in a clinical response in two-thirds of patients, with an acceptable safety profile
Am J Gastroenterol 2024 [59]	Comparative study	Side-effects of vedolizumab versus anti-TNF	Vedolizumab was associated with increased risks of serious infections compared with anti-TNF in CD but not in UC.
Gastroenterology 2018 [60]	Comparative study in IBD patients in France	Infections in IBD	An heterogeneity in risks of serious and opportunistic infections in IBD patients treated with immune-suppressive regimens was found.
Inflamm Bowel Dis	Observatio-nal study	Ustekinumab in CD	SC ustekinumab is an effective treatment option for maintaining long-

2017<br>[63]			term response in patients with CD failing anti-TNF therapy
Clin Gastroenterol Hepatol<br>2022<br>[64]	Randomized controlled trial	Maintence treatment with ustekinumab	Patients receiving subcutaneous ustekinumab maintained clinical remission through 5 years.
Inflamm Bowel Dis<br>2023<br>[65]	Clinical trial	Side-effects of tofacitinib	Most Herpes zoster events were mild to moderate. Herpes zoster incidence rates remained stable over 7.8 years of exposure.
J Gastrointest Surg<br>2024<br>[66]	Review	Surgery for IBD	Surgical treatment of UC and CD from the gastroenterologist's stand-point.
Dig Dis Sci<br>2024<br>[69]	Review	Polypharmacy in older patients with IBD	Polypharmacy is prevalent in older adults with IBD and independently associated with low quality of life.
Am J Gastroenterol<br>2024<br>[70]	Review (Nationwide cohort)	Analgesics in older IBD patients	Older adults most commonly received analgesic prescriptions within 1 year after IBD diagnosis.
